# Fabrication and characterization of PVA/NNSA/GLA/nano-silica proton conducting composite membranes for DMFC applications

**DOI:** 10.1080/15685551.2019.1626323

**Published:** 2019-06-06

**Authors:** Sajede Shabanpanah, Abdollah Omrani, Moslem Mansour Lakouraj

**Affiliations:** Faculty of Chemistry, University of Mazandaran, Babolsar, Iran

**Keywords:** Hybrid membrane, poly (vinyl alcohol), proton conductivity, methanol permeability

## Abstract

Blends of PVA and 2-nitroso-1-naphtol-4-sulfonic acid (NNSA) ranging from 10 to 40 wt% were crosslinked in the presence of glutaraldehyde (GLA) to produce hybrid membranes. The structure and morphology of the hybrid membranes were studied by XRD, FE-SEM, EDX, and elemental mapping experiments. The mechanical performance and thermal stability of the membranes were also examined by dynamic mechanical analysis (DMA) and thermogravimetry analysis (TGA), respectively. Increasing the concentration of NNSA resulted in the improvement of mechanical and thermal performances of the membrane. The addition of NNSA and SiO_2_ to the solution of PVA makes the resultant hybrid membrane more hydrophilic, and therefore, the proton conductivity, water uptake and ion exchange capacity (IEC) improved. The highest proton conductivity value (0.18 S cm^−1^ at 30 °C) was found for the PVA/GLA/NNSA (40 wt%)/SiO_2_ (5 wt%) composite membrane. It was also demonstrated that the methanol permeability values decreased with increasing NNSA content.

## 1. Introduction

Fuel cells convert chemical energy directly into electricity, and therefore, have a wide range of applications. The direct methanol fuel cell (DMFC) [1–4] and solid polymer electrolyte membranes fuel cell (PEMFC) [5–7] have recently been the focus of many researches because of some advantages, such as high-energy efficiency and low emission of pollutants. The PEMFCs have been used in aerospace, transportation, and mobile power stations [8–10]. Over the past few decades, the perfluorosulfonate ionomer membranes, such as Nafion membranes, have been the most commonly utilized polymer membrane for DMFC applications. Although the Nafion membranes show high chemical stability and good proton conductivity [11,12], they have a high fuel permeation with a high cost [13,14]. Also, the methanol permeation can reduce the system performance. To overcome these drawbacks, we have to look for suitable alternatives to Nafion membranes. Recently, the development of Poly (vinyl alcohol) (PVA) membranes for DMFC applications are of interest because PVA is a biodegradable semi-crystalline synthetic polymer with good film forming capability, good methanol barrier, excellent thermal and chemical stabilities, and being non-toxic [15–18]. However, a problem for the PVA-based membranes is their low proton conductivity due to the fact that PVA does not have any charge functional groups, such as carboxylic (-COOH) or sulfonic acid (-SO_3_H) groups. As a result, the PVA with functional groups should be provided in order to be used as PVA polymer membranes for DMFC applications. Therefore, the use of an acid group, as a donor, is one of the suitable solutions for increasing the conductivity of PVA membranes [19]. For instance, Rhim et al. [20] studied the proton conduction of the PVA/sulfosuccinic acid (SSA) membrane in which the SSA acts as a proton donor and a cross-linker agent. These cross-linked PVA/SSA membranes had the proton conductivity in the range of 10^−3^ to 10^−2^ S/cm and showed a reduced methanol permeability than Nafion 117. Kim et al. [21] prepared the PVA/sulfosuccinic acid (SSA)/silica membranes containing different contents of SSA with methanol permeability and proton conductivity in the range of 10^−8^ − 10^−7^ cm^2^ S^−1^ and 10^−3^–10^−2^ S cm^−1^, respectively. In another study, Kim et al. [22] reported a novel cross-linked PVA/poly (styrene sulfonic acid-co-maleic acid) (PSSA-MA)/clay membrane with excellent resistance to methanol permeation (2.19 × 10^−7^ cm^2^ s^−1^) and good proton conductivity (0.023 S cm^−1^). Gomes and Filho [23] synthesized some hybrid membranes using PVA, phosphotungstic acid and diethylenetriamine penta acetic acid with the maximum conductivity of 8.59 × 10^3^ S cm^−1^. Some researchers have recently used the double-layer membranes based on sulfonated poly (ether ether ketone)/poly (vinyl alcohol) (SPEEK/PVA blend) with high mechanical stability and low swelling ratio [24,25]. Interestingly, Kumar et al. [26] prepared the poly (vinyl alcohol)/para toluene sulfonic acid (PVA/PTSA) polymer membrane for a DMFC. They chose suitable concentrations of the proton carriers (-SO_3_H groups) to increase the ionic conductivity of PVA/PTSA membrane. The 10 wt % PVA/10 wt % PTSA membrane showed a selectivity of 12.7 × 10^7^ m S cm^−3^, which is higher than that of Nafion 117 membrane. It is obvious that the pure PVA has a low stability in aqueous solutions that can limit its use. One way to increase the stability of PVA is its crosslinking.

The purpose of this study is to prepare some PVA hybrid membranes with good conductivity, low permeability of methanol, and good stability. In this regard, we modified the PVA membrane with 2-nitroso-1-naphtol-4-sulfonic acid (NNSA) to increase its conductivity. Also, an attempt was made to find a relationship between the content of NNSA and the properties studied. Glutaraldehyde (GLA) as a cross-linker was added to the system to maintain the membrane stability. Also, the role of non-toxic and hydrophilic silica nanoparticles was evaluated.

## 2. Experimental

### 2.1. Materials

Poly (vinyl alcohol) (PVA, Mw = 27,000 g/mol) and 2-nitroso-1-naphtol-4-sulfonic acid were purchased from Fluka. Glutaraldehyde (25 wt % solution in water) and SiO_2_ nanoparticles (average particle size 10–20 nm) were supplied from Sigma-Aldrich. Nafion 117 (perfluorinated membrane) was provided by Sigma-Aldrich and used as received. Methanol, sodium hydroxide, sodium chloride, and sulfuric acid were all obtained from Merck. Deionized water was used in all the experiments.

### 2.2. Membrane preparation

The PVA hybrid membranes were prepared at different concentrations of NNSA by a solution-casting method. At first, a certain amount of PVA was dissolved in deionized water at 60 °C under stirring to provide a 10 wt % solution. Then, the appropriate amounts of 2-nitroso-1-naphtol-4-sulfonic acid (10, 20, 30 and 40 wt %) were mixed with the PVA solutions under stirring for 4 h. The SiO_2_ nanoparticles (5 wt %) were dispersed in the above mixture under ultrasonic treatment for 2 h. Glutaraldehyde, as a cross linker, was also added under acidic conditions at 40 ^◦^C. The trapped air in the resultant transparent and viscous mixture was removed under vacuum, and then, the solutions were poured into plastic Petri dishes and placed at ambient temperature until the complete water evaporation. The final thickness of the dried membrane was between100 and 200 µm.

### 2.3. Membrane characterization

The attenuated total reflectance infrared (ATR-IR) spectroscopy (Thermo Nicolet, model Avatar 370) was used to obtain some information on the functional groups present in the membranes. The surface morphology, elemental composition, and X-ray mapping of the membranes were studied by a field emission scanning electron microscopy (FE-SEM, Model Mira 3-XMU) equipped with an energy dispersive spectroscopy (EDS). The crystal structures of the PVA membranes were investigated using a Philips PW1730 X-ray diffractometer with Cu Kα radiation of wavelength λ= 1.5418 Å for 2θ angles between 10° and 80°. The thermal stability of the membranes was examined using a thermogravimetric analyzer (BÄHR Thermoanalyse GmbH STA 450, Germany) at the heating rate of 10 ^◦^Cmin^−1^ in the temperature range of 25–600 ^◦^C. Dynamic mechanical thermal analyses (DMTA) were conducted using a Tritec 2000 DMA-Thermal analyzer (Triton Technology Co., England) at a frequency of 1 Hz and oscillation amplitude of 0.18 mm. The DMTA measurements were carried out from 25 to 150 ^◦^C under air atmosphere at a heating rate of 10 ^◦^Cmin^−1^.

### 2.4. Water uptake measurements

The water uptake of the membranes was examined through the assessment of the mass change before and after the water absorption. Accordingly, a piece of membrane was immersed in deionized water for a day, and then, after removing water from the surface of the membrane, the sample was quickly weighed. Afterwards, the specimen was dried in a vacuum oven at 60 ° C. The water uptake (WU), was calculated as:

(1) $$WU\left(\%\right)=\frac{W_{wet}-W_{dry}}{W_{dry}}\times 100\%$$

Where, W_wet_ and W_dry_ are the mass of fully hydrated membrane, and of the dry membrane, respectively.

### 2.5. Ion-exchange capacity

The ion exchange capacity (IEC) of the PVA membranes was evaluated using the titration technique. At first, the samples are placed in deionized water. Then, they were immersed in a large volume of HCl solution to fully protonate the acid functionalities. After washing with water to remove the remaining excess acid, they were equilibrated with 20 ml of 2 M NaCl solution for 48 h to release H^+^ ions into solution. The released H^+^ ions were titrated using a standard 0.01 M NaOH solution, as the titrant, and phenolphthalein, as the indicator.

### 2.6. Conductivity

**The proton conductivities of the membranes were measured using** a frequency response detector (EG&G model 1025), which runs under the control of M 398 software. The measurements were performed at a temperature range of 30–70 ^◦^C over the frequency range 0.005 Hz −100 kHz with the voltage amplitude of 10 mV. Each sample was cut in the approximate sizes of 1 cm × 1 cm, and soaked in the deionized water for 12 h prior to being mounted on the cell. The membranes were sandwiched between two brass plates acting as anode and cathode electrodes. The proton conductivity (σ) was obtained as:

(2) $$\sigma =\frac{L}{RA}$$

Where, σ is the proton conductivity (in S/cm), L is the membrane thickness (in cm), R is the resistance obtained from the impedance (in Ω), and A is the cross-sectional area of the membrane (in cm^2^). The impedance of each sample was measured three-times.

### 2.7. Methanol permeability test

Resistance to methanol crossover of the membranes was measured using a glass diffusion cell. The membrane was clamped between two compartments A and B. The compartment A was loaded with 20 mL CH_3_OH aqueous solution (2M) and the compartment B was loaded with 20 mL deionized water. In each compartment, a magnetic stirrer was used to ensure the uniform concentration of the compounds. The concentration of the permeating methanol was then detected using gas chromatography equipped with a thermal conductivity detector (GC-7890A with a DB-5 column, Agilent, USA). The methanol concentration in the receiving compartment as a function of time is given by

(3) $$P=\;\frac{1}{A}\;\frac{C_{B}\left(t\right)}{C_{A}\left(t-t_{0}\right)}\;V_{B}L$$

Where, C_A_ and C_B_ are the concentrations of methanol in compartments A and B, respectively, V_B_ is the volume of the permeated compartment, and A and L are the area and thickness of the membrane, respectively.

## 3. Results and discussion

### 3.1. FTIR spectra

**Figure 1.**
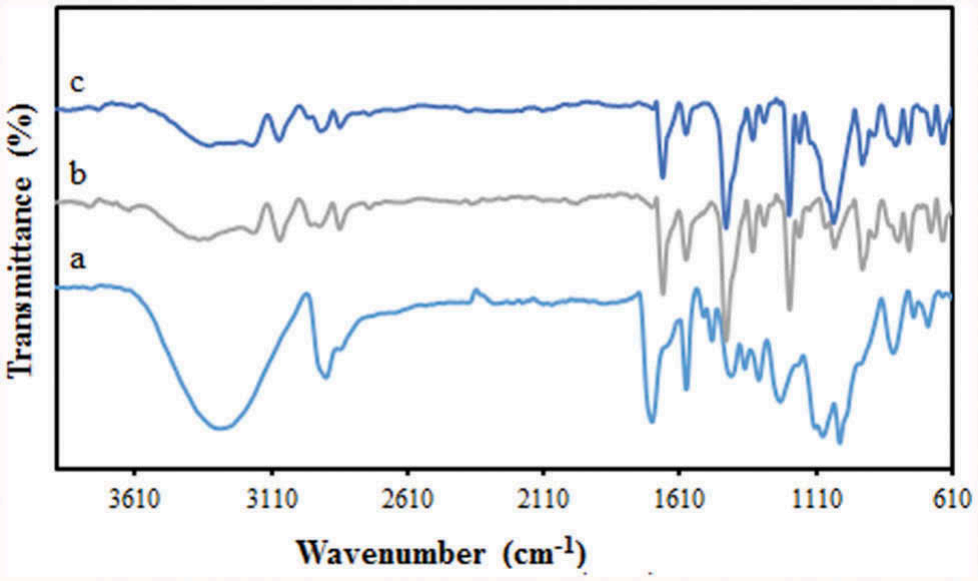
FTIR spectra of the PVA hybrid membrane (a) pure PVA (b) PVA/GLA/10 wt % NNSA membrane and (c) PVA/GLA/40 wt % NNSA/5 wt % SiO_2_ membrane.

FTIR spectroscopy was utilized for the identification of the functional groups involved in the synthetic membranes. The FTIR spectrum of pure PVA (Figure 1(a)) shows the characteristic PVA spectrum in agreement with the literature [27] The broad peak at around 3000 and 3400 cm^−1^ corresponds to the stretching vibration of O–H present in the intramolecular and intermolecular hydrogen bonds. The peaks appeared at 2840 cm^−1^ and 2920 cm^−1^ are respectively attributed to the symmetric and antisymmetric stretching vibrations of alkyl C–H bonds. After reaction with the GLA (Figure 1(b)), the bands at 1250–1270 cm^−1^ are attributed to the ether bonds (C-O-C) formation by the reaction between CHO groups of the cross-linker and OH groups of the PVA [28] Also, after cross-linking with GLA, the intensity of the O–H stretching vibration band is reduced [29] The presence of the sulfonic acid group in the membrane was confirmed by the characteristic asymmetric and symmetric S = O stretching vibrations at 1030 and 1145 cm^−1^, respectively. The absorption band at 830 cm^−1^ identifies the existence of the S–O stretching of SO_3_H groups [30]. In addition, the observed bands at around 800 and 1050 cm^−1^ are characteristic of Si-O-Si symmetric and asymmetric vibrations, respectively [31].

### 3.2. SEM observations

The surface morphologies of the prepared membranes were shown in Figure 2. The SEM images revealed the presence of SiO_2_ nanoparticles in the PVA matrix. The homogenous and smooth morphology shown in Figure 2(a) is related to the pure PVA. From the SEM photographs displayed in Figure 2(b–f), it was demonstrated that the morphology of the hybrid membrane could be influenced considerably by the presence of NNSA, silica nanoparticles and crosslinking reaction. Five SEM images were shown at different magnifications and in both the micrometer and nanometer scales to better comparison of the raised morphology and to confirm the development of a needle type morphology after crosslinking of PVA by GLA. However, FE-SEM images of the hybrid membrane unveil a relatively uniform distribution of the NNSA and SiO_2_ nanoparticles throughout the PVA matrix with increased mechanical stability. The presence of Si, N and S elements were confirmed using energy-dispersive X-ray analysis (EDXA) (Figure 2(g)). In fact, Figure 2(g) illustrates the incorporation of the sulfonic salt and silica nanoparticles in the cross-linked PVA. Also, the EDXA mapping, as shown in Figure 3, shows the uniform distribution of silica nanoparticles over the surface of the membrane.

**Figure 2.**
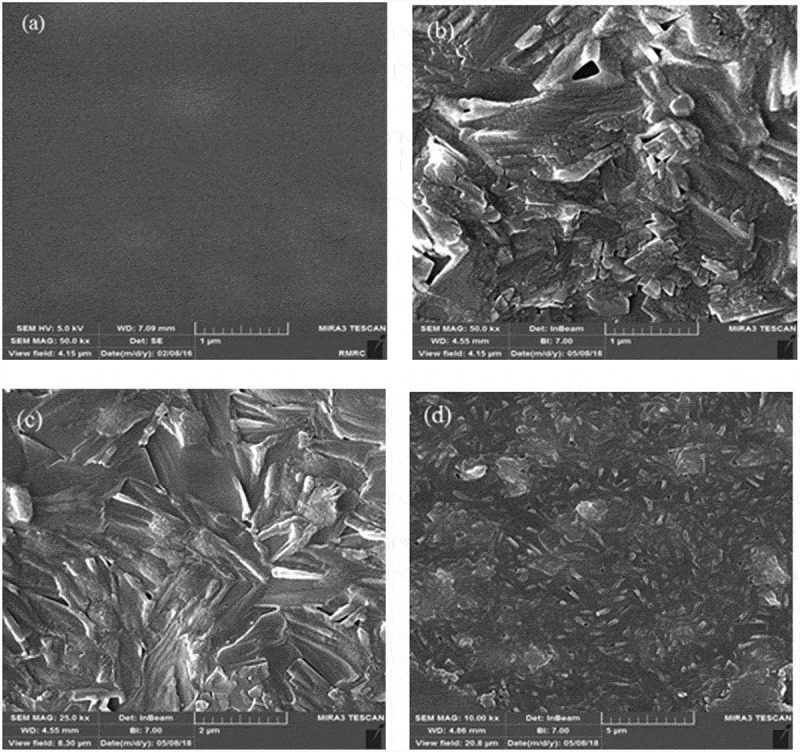
SEM photographs for (a) pure PVA (b-f) PVA/GLA/NNSA (40 wt%)/SiO_2_ (5 wt%) hybrid membrane at various magnifications and (g) EDXA of the hybrid membrane.

**Figure 2.**
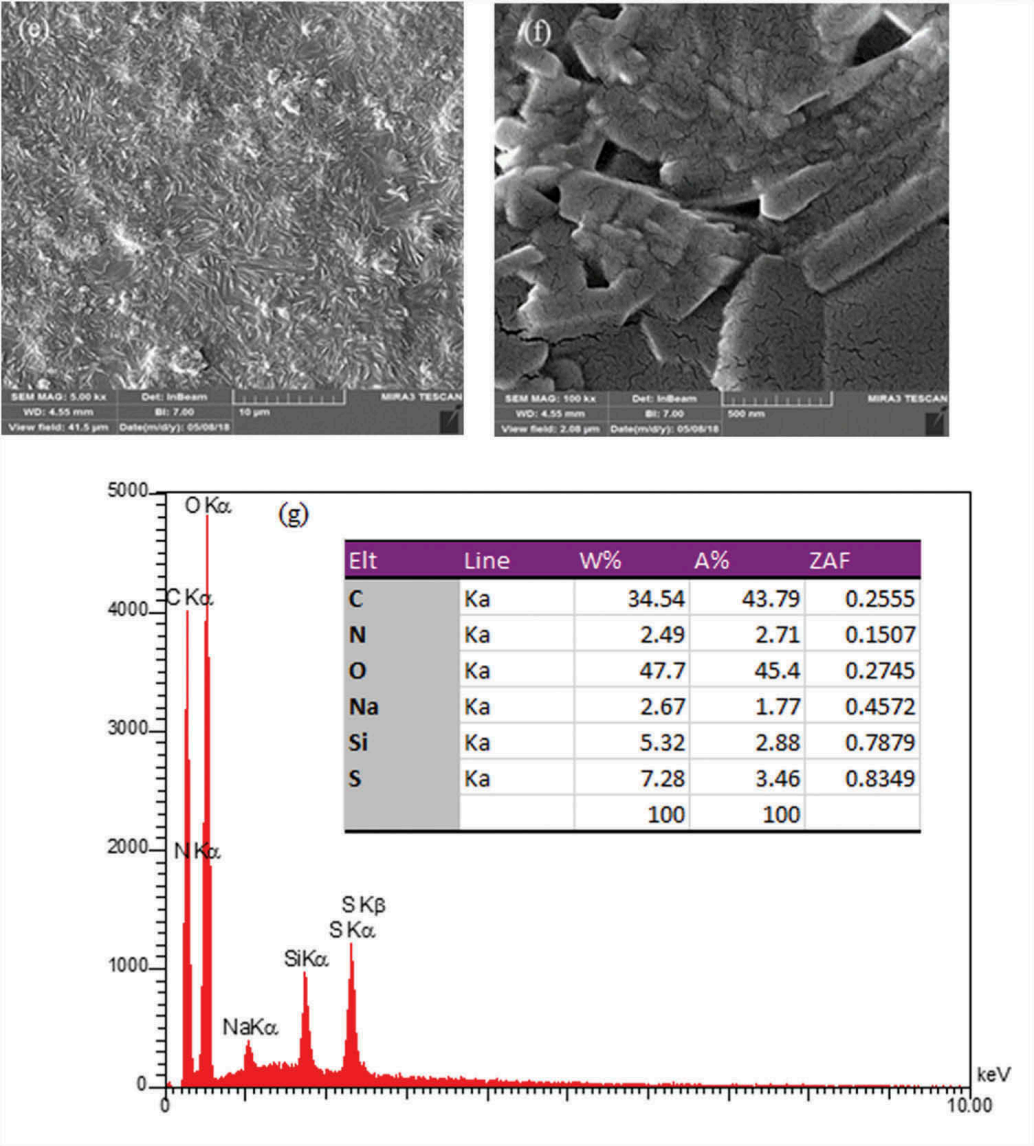
(Continued).

**Figure 3.**
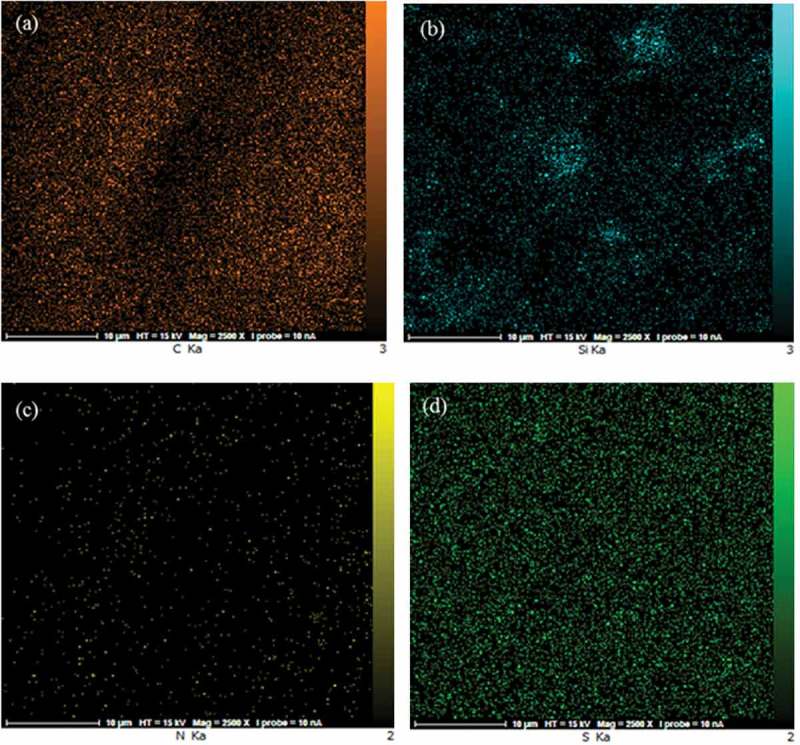
Dispersion of (a) C, (b) Si, (c) N and (d) S in the surface of the PVA membranes.

### 3.3. XRD results

**Figure 4.**
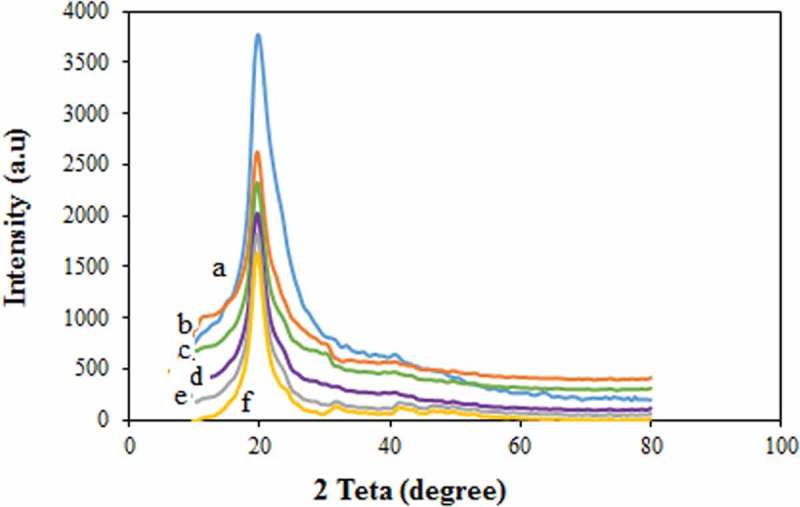
XRD patterns of the prepared membranes (a) pure PVA, (b-e) PVA/GLA/10,20,30,40 wt % NNSA and (f) PVA/GLA/40 wt % NNSA/5 wt % SiO_2._

The crystallinity is one of the major factors that affects the mechanical properties of a polymer. The XRD analysis was carried out to study the crystallinity of the PVA membranes. The XRD curves of the PVA and modified PVA membranes are presented in Figure 4. Accordingly, the pure PVA membrane has a semi-crystalline structure with a large peak at a 2θ angle of 19–20^◦^ [32,33]. The peak intensity of XRD for the modified PVA membrane was reduced, which can be indicative of the increase of the amorphous phases existed in the PVA/GLA/NNSA/SiO_2_ membrane. Also, the intensity of the modified PVA diffraction peak decreased with increasing the NNSA content. Therefore, it can be said that the degree of the amorphous structure of the membranes increases because of the addition of the cross-linker agent and the interactions between the silica nanoparticles and NNSA as well.

### 3.4. Water uptake studies

**Figure 5.**
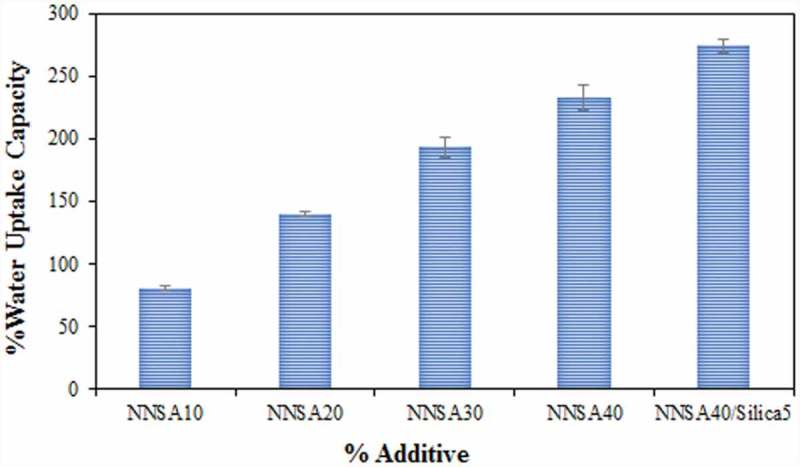
The change of water uptake in the PVA membranes.

Water uptake has an effective role in determining the properties of the membrane. The water uptake of the composite PEMs as a function of NNSA contents is shown in Figure 5. Increasing the NNSA content led to the increase of water uptake, which is related to water retention due to the hydrophilic nature of the available functional groups in the modified PVA. These results indicate that the increase of certain surface areas of the membrane structure leads to higher water retention. The reduction of crystallinity and stronger interactions between the absorbed water and the network of the PVA/GLA/NNSA/SiO_2_ membrane enhance the proton conductivity. In fact, when the membrane keeps more water, the number of exchange sites available per cluster increases, and therefore, the proton conduction rate increases [34].

### 3.5. Ion exchange capacity assessment

**Figure 6.**
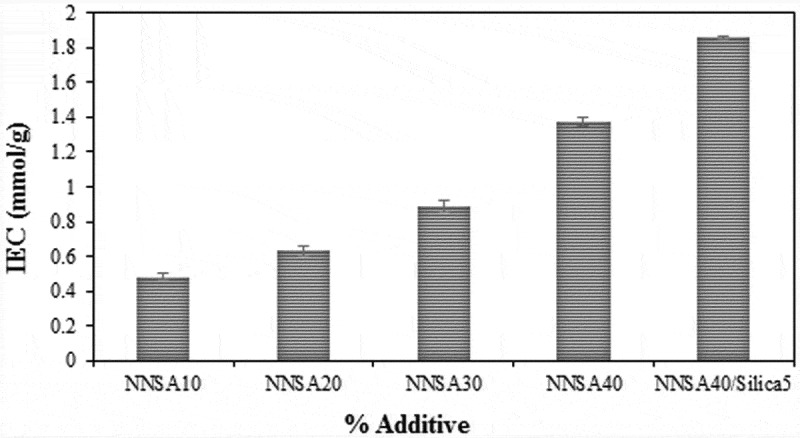
The change of IEC values in the PVA membranes.

The ion exchange capacity (IEC) data of the PVA hybrid membranes are shown in Figure 6. The IEC values gradually increased from 0.48 to 1.86 mmol/g with increasing the NNSA concentration, which can be because of the increased charged groups in the membranes. These results are similar to the trend of water absorption and conductivity experiments because the increase of water absorption provides an appropriate space for the proton transfer mechanisms [35]. Therefore, the addition of the sulfonic acid groups leads to more hydrophilic membranes and the exchange rate of the ion is facilitated. However, the use of highly hydrophilic membranes decreases the system stability relative to water or methanol. Hence, the content of NNSA and cross-linker agent should be controlled in the system.

### 3.6. Conductivity measurement

**Figure 7.**
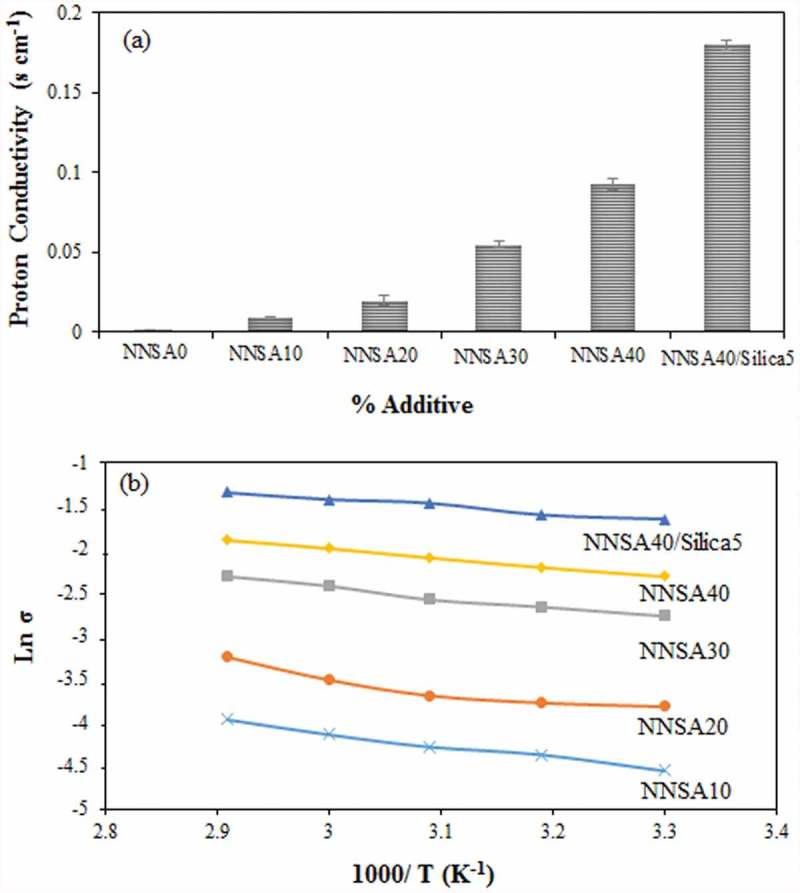
(a) Proton conductivity of the hybrid membranes and (b) Arrhenius plot of PVA hybrid membranes.

Proton conductivity is an important parameter used to investigate the tendency of the membrane to proton transport. The proton conductivities of the cross-linked PVA membranes with different concentrations of NNSA are shown in Figure 7(a). The proton conductivities of the PVA membranes were in the range of 10^−4^ to 10^−1^ S/cm at room temperature. Apparently, conductivity is a function of NNSA content and increases sharply with the increase of the sulfonic acid group. The increase in the proton conductivity of the membrane can be attributed to the presence of sulfonic acid groups (–SO_3_^−^H^+^) as a proton donor in the modified PVA membranes. This result agrees with the IEC and water uptake assessments. Two principle mechanisms are known for the proton transfer. The first mechanism is the Grotthus mechanism, the proton diffusion with the bound water, which is considered as the proton jump from one solvent molecule to the next one via a hydrogen bond. The second mechanism is the vehicle mechanism, the proton diffusion with the free water, which assumes that the proton diffuses together with the solvent molecules by forming a complex (i.e., H_3_O^+^) and then diffuses intact. The activation energy, defined as the minimum energy required for proton transport, was obtained using Equation (4):

(4) $$\ln\sigma =-\frac{E_{a}}{RT}$$

Where, σ is the proton conductivity (in S/cm), E_a_ is the activation energy (in kJ/mol), R is the universal gas constant (= 8.314 J/(mol K)), and T is the absolute temperature (in K).

As shown in Figure 7(b), the ln σ depends almost linearly on 1/T. The E_a_ values of five PVA membranes are shown in Table 1. The apparent activation energy (E_a_) for the proton conduction decreased slightly with the increase of the NNSA concentration. Also, it was found that the process of proton transfer facilitates at higher temperatures. These observations could be described by an increase in the hydrogen bonding and a decrease in the content of free water of the hybrid membranes. Moreover, the presence of SiO_2_ nanoparticles provided more proton hopping sites leading to higher proton conductivity and lower activation energy. As shown in Table 1, the value of *E*_a_ for the synthesized membranes changes between 6.43 to 12.14 kJ/mole. These values are close to 10 kJ mol^−1^, indicating that both the Grotthuss and vehicle mechanisms have contributions simultaneously for the proton transfer. However, the Grotthuss mechanism becomes more predominant, in which the ion transport proceeds through the hydrogen bonding.

**Table 1.** Apparent activation energy, Ea, of proton conduction for the cross-linked PVA membranes.

Membrane	% Additive	E_a_/kJ mol^−1^
PVA-GLA-NNSA	NNSA10	12.14
PVA-GLA-NNSA	NNSA20	11.90
PVA-GLA-NNSA	NNSA30	9.75
PVA-GLA-NNSA	NNSA40	8.98
PVA-GLA-NNSA-SiO_2_	NNSA40-SiO_2_ 5	6.93

### 3.7. Methanol permeability measurement

**Figure 8.**
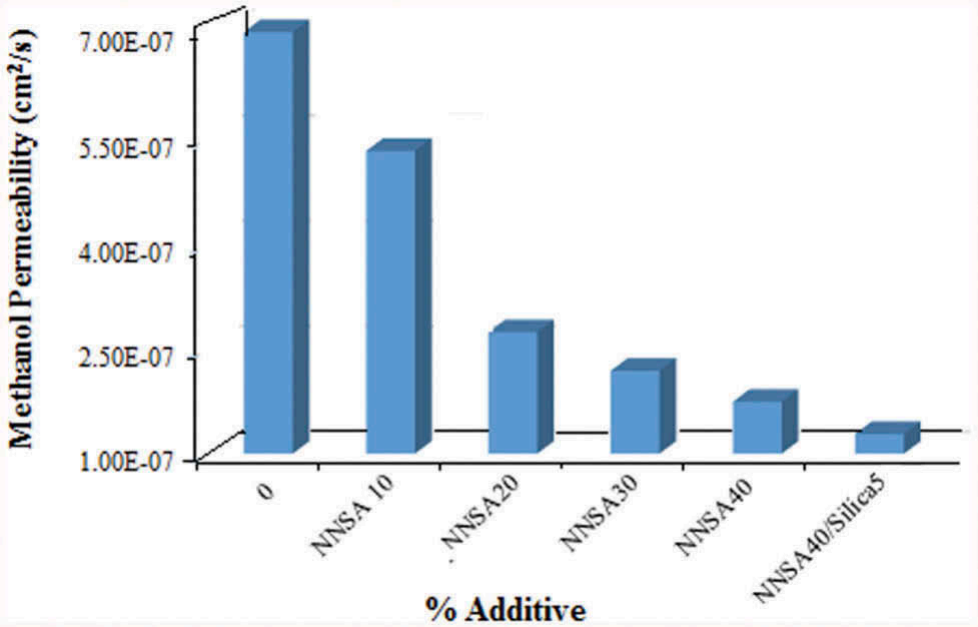
Methanol permeability of PVA membranes.

Another effective factor in a proton exchange membrane (PEM) is having low methanol permeability if methanol is used as fuel. Resistance to methanol crossover of the membranes were calculated using Equation (3), and the results were shown as a function of NNSA concentration in Figure 8. The methanol permeability of Nafion 117 evaluated under the same experimental conditions, which was determined as 1.83 × 10^−6^ cm^2^/sec, was in good agreement with the value reported in the literature [20] The methanol permeability of all the cross-linked PVA membranes is lower than that of Nafion 117. The methanol diffusion coefficient was found to be 5.31 × 10^−7^ cm^2^/s for the membrane containing 10 wt % NNSA and decreased continuously with increasing the NNSA content reaching a value of 1.49 × 10^−7^ cm^2^/s for the membrane with 40 wt % NNSA/5 wt % SiO_2_. This result can be explained on the basis of the chemical interaction between methanol and the ionic clusters introduced in the modified membranes. In fact, NNSA and SiO_2_ moieties may interfere with methanol permeation in the above mechanism, while improves proton transport through the sulfonic acid groups. Therefore, it is expected that the methanol permeability should be decreased because the silica nanoparticles and NNSA act as a cluster for blocking the methanol transport.

### 3.8. Thermal stability of the membranes

**Figure 9.**
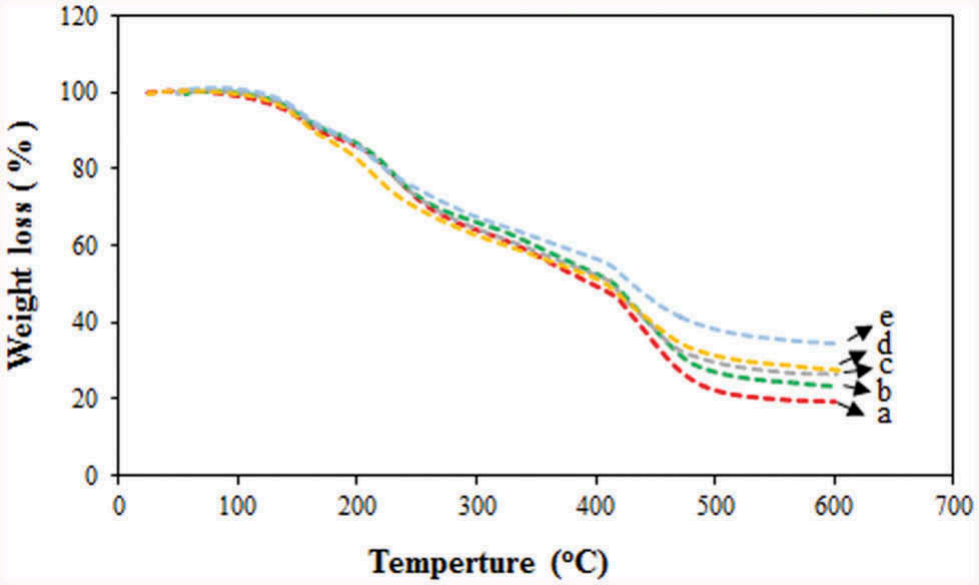
TGA curves of PVA hybrid membranes (a-d) PVA/GLA/10,20,30,40 wt % NNSA and (e) PVA/GLA/40 wt % NNSA/5 wt % SiO_2._

The effect of different contents of NNSA on the thermal stability of the hybrid membranes was investigated by TGA (Figure 9). Accordingly, a multi-stage TGA curve was seen for all the membranes. The first stage was observed at around 100°C, attributed to the loss of adsorbed water by the hygroscopic property of the membrane [36] The second stage around 180ºC corresponds to the degradation of GLA and PVA in the polymer membranes [37] The third stage at 280°C was attributed to the degradation of sulfonic acid groups in the membrane which becomes more pronounced with increasing the NNSA content. The fourth stage is related to the degradation of backbones of cross-linked membranes [38]. The weight residue gradually increased as the NNSA content increased. Therefore, the incorporation of NNSA into the cross-linked PVA polymer significantly improved the thermal stability of the membranes. The higher residual mass was achieved by the PVA/GLA/40 wt % NNSA/5 wt % SiO_2_ membrane as compared to the other four membranes.

In fact, it can be said that the interaction between particles in the polymer matrix provide a rigid structure with a higher thermal resistance. Also, the presence of silica nanoparticles at the highest NNSA concentration provides strong interactions with the polymer matrix leading to the improved thermal stability.

### 3.9. Mechanical properties

**Figure 10.**
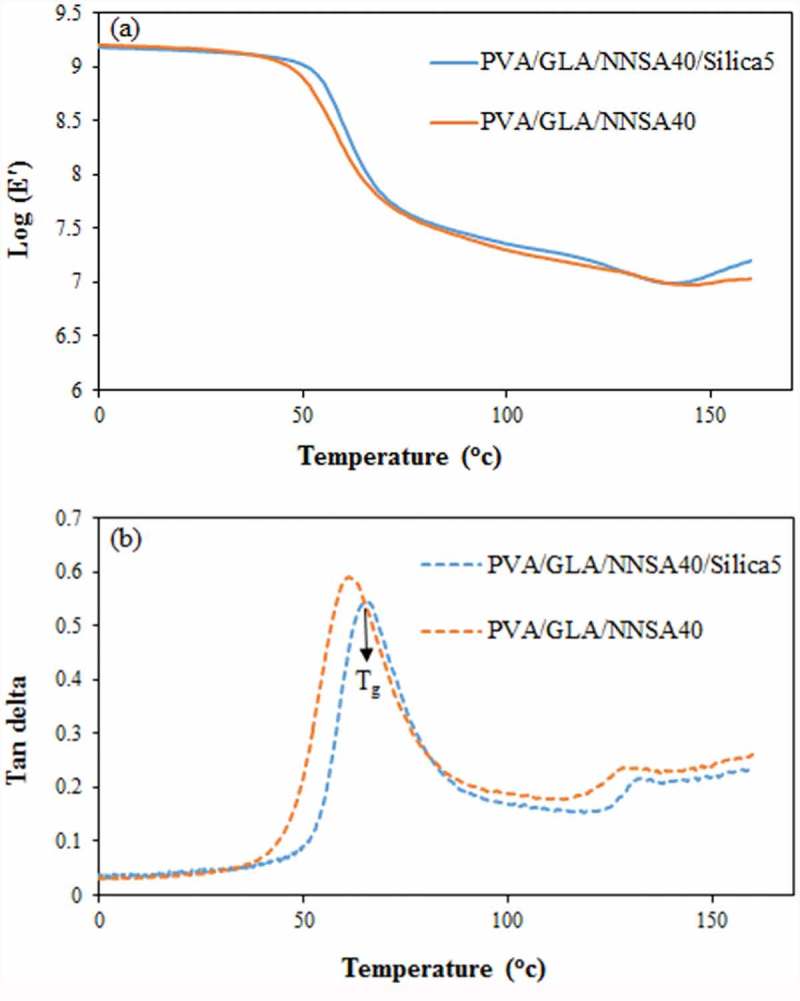
The DMA curve for the PVA hybrid membranes (a) storage modulus curve (b) tan (δ) curve.

The DMTA imposes a sinusoidal stress on a sample in the three-point bending mode and determines the sample modulus and tan δ as a function of temperature or frequency. Figure 10(a) shows the variation of storage modulus (E′) vs. temperature for the PVA hybrid membranes. The storage modulus of the PVA/GLA/NNSA 40 wt % membrane (E′ = 1.36 × 10^9^ dyn cm^−2^) was lower than that of the PVA/GLA/NNSA 40 wt %/5 wt % SiO_2_ membrane (E′ = 1.38 × 10^9^ dyn cm^−2^) at 30°C. It was also confirmed that the SiO_2_ nanoparticles enhanced the mechanical properties of the PVA membranes. Figure 10(b) shows the loss factor or tan (δ) vs. temperature curves of the PVA membranes. The first tan (δ)_1_ peak shows the glass transition temperatures (T_g_) of 62 and 65 °C for the PVA/GLA/NNSA 40 wt % and PVA/GLA/NNSA 40 wt %/5 wt % SiO_2_, respectively. The decrease of T_g_ upon addition of silica nanoparticles is related to the lower degree of crystallinity of the PVA hybrid membrane [19]. The second tan (δ)_2_ peak may be due to the slip of the PVA molecular chains at around 130 and 132°C. The intensity reduction of the tan (δ) peaks may be due to the decrease of the degree of crystallinity and increase of the stiffness of the hybrid membrane upon addition of silica nanoparticles.

## 4. Conclusions

The PVA/GLA/NNSA membranes were successfully fabricated by a solution casting method. The thermal and mechanical properties of the hybrid membranes were improved by increasing the NNSA content. Also, the polymer networking with a cross-linker agent formed a rigid membrane whose resistance to mechanical and thermal effects increased. The thermal stability of the membranes was also improved in the presence of silica nanoparticles. Water uptake and proton conductivity of the membranes increased with addition of the sulfonic acid group, which increases the hydrophilicity of the system and thus facilitates the transfer of the proton. Methanol permeability of the PVA hybrid membranes decreased with the NNSA loading in the presence of the silica nanoparticle. From a practical point of view, the results obtained from this study can be used for further development of hybrid polymer membranes to be applied in direct methanol fuel cell.
